# Introgression of Ivermectin Resistance Genes into a Susceptible *Haemonchus contortus* Strain by Multiple Backcrossing

**DOI:** 10.1371/journal.ppat.1002534

**Published:** 2012-02-16

**Authors:** Elizabeth Redman, Neil Sargison, Fiona Whitelaw, Frank Jackson, Alison Morrison, David Jon Bartley, John Stuart Gilleard

**Affiliations:** 1 Institute of Infection, Immunity and Inflammation, College of Medical, Veterinary and Life Sciences, University of Glasgow, Glasgow, United Kingdom; 2 Moredun Research Institute, Pentlands Science Park, Midlothian, United Kingdom; 3 Department of Comparative Biology and Experimental Medicine, Faculty of Veterinary Medicine, University of Calgary, Calgary, Alberta, Canada; McGill University, Canada

## Abstract

Anthelmintic drug resistance in livestock parasites is already widespread and in recent years there has been an increasing level of anthelmintic drug selection pressure applied to parasitic nematode populations in humans leading to concerns regarding the emergence of resistance. However, most parasitic nematodes, particularly those of humans, are difficult experimental subjects making mechanistic studies of drug resistance extremely difficult. The small ruminant parasitic nematode *Haemonchus contortus* is a more amenable model system to study many aspects of parasite biology and investigate the basic mechanisms and genetics of anthelmintic drug resistance. Here we report the successful introgression of ivermectin resistance genes from two independent ivermectin resistant strains, MHco4(WRS) and MHco10(CAVR), into the susceptible genome reference strain MHco3(ISE) using a backcrossing approach. A panel of microsatellite markers were used to monitor the procedure. We demonstrated that after four rounds of backcrossing, worms that were phenotypically resistant to ivermectin had a similar genetic background to the susceptible reference strain based on the bulk genotyping with 18 microsatellite loci and individual genotyping with a sub-panel of 9 microsatellite loci. In addition, a single marker, Hcms8a20, showed evidence of genetic linkage to an ivermectin resistance-conferring locus providing a starting point for more detailed studies of this genomic region to identify the causal mutation(s). This work presents a novel genetic approach to study anthelmintic resistance and provides a “proof-of-concept” of the use of forward genetics in an important model strongylid parasite of relevance to human hookworms. The resulting strains provide valuable resources for candidate gene studies, whole genome approaches and for further genetic analysis to identify ivermectin resistance loci.

## Author Summary

The use of drugs (anthelmintics) to control nematode parasites (roundworms) is common in both humans and animals. This has led to the widespread development of drug resistance in livestock parasites and serious concerns regarding its emergence in human parasites. *Haemonchus contortus* is a parasitic nematode of sheep that has a high propensity to develop resistance and is the most widely used model system in which to study anthelmintic drug resistance. Ivermectin is an extremely important drug for parasite control in both humans and animals. Here, we report a novel approach using genetic crossing to transfer a region of the *H. contortus* genome containing ivermectin resistance genes from resistant strains into a susceptible strain. During our backcrossing approach, we have identified a genetic marker showing evidence of genetic linkage to ivermectin resistance. The susceptible strain we have used is currently having its complete genome sequenced making the information and strains generated here extremely valuable for the identification of ivermectin resistance genes. This work represents an important proof of concept for using genetic approaches to identify genomic regions containing drug resistant genes in parasitic nematodes.

## Introduction

Parasitic nematode worms are important human and animal pathogens. Human parasites infect well over 1 billion people worldwide and livestock parasites cause major economic production loss to grazing ruminants. Control is dependent on the use of a limited number of anthelmintic drugs and intensive use of these has already led to widespread resistance in livestock parasites [1]–[5]. In recent years selection pressure has been applied to parasitic nematode populations in humans by anthelmintic treatments used in various control programs and, in the case of some filarial nematodes, eradication programs [6]. In endemic regions, parasitic nematodes often occur as mixed species infections and so the application of drug treatments to control one parasite species inevitably leads to selection pressure being applied to others. Consequently, there is increasing concern about the development of anthelmintic drug resistance in nematode parasites of humans.

Unfortunately, parasitic nematodes of humans make extremely difficult experimental subjects and so there is a need to develop model systems to study potential mechanisms of anthelmintic resistance. *Haemonchus contortus* is a parasitic nematode of sheep which has a high propensity to develop anthelmintic. It is also one of the most amenable parasitic nematodes to experimental manipulation which, together with recent progress in sequencing its genome makes it a potentially powerful model system to study drug resistance in the strongylid nematode group [7]. In addition, genetic crossing is technically possible in this parasite and has potentially powerful applications in the study of anthelmintic resistance providing we can develop the necessary techniques and resources [8]–[11].

The genetic basis of anthelmintic resistance is still relatively poorly understood. To date, most research has focussed on the investigation of possible associations between the resistance phenotype and polymorphisms in candidate genes. This approach has been successful in identifying polymorphisms in the isotype-1 β-tubulin gene as important determinants of benzimidazole resistance [12]–[13]. However, candidate gene studies have major limitations and have yet to unequivocally identify molecular loci responsible for resistance against other anthelmintic classes [14]. Genomic resources are improving for many parasitic nematodes, including the production of high quality reference genome sequences, which will allow the application of genome-wide approaches that do not depend on prior assumptions regarding potential resistance mechanisms [14]–[19]. However, the application of such approaches is not likely to be a trivial task. Attempts to associate specific genetic differences with a drug resistant phenotype will be complicated by the high level of genetic variation that often exists within and between parasitic nematode populations [20]–[22]. Simple comparisons will potentially reveal many genetic differences between drug resistant and susceptible parasite strains that are not necessarily associated with anthelmintic resistance but with background genetic variation or with other unrelated phenotypes. Consequently, there is a need to develop experimental approaches to overcome these challenges.

**Figure 1:**
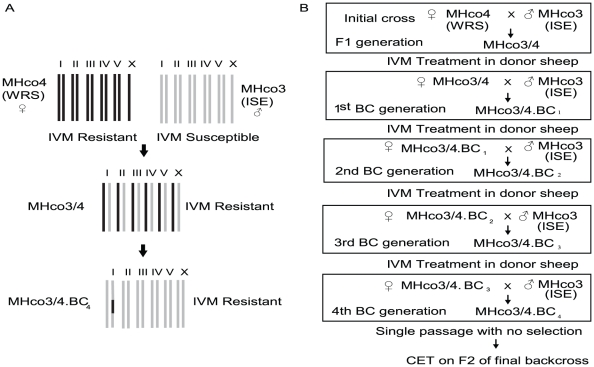
Backcrossing approach to introgress ivermectin resistance-conferring genes from resistance strains into the MHco3(ISE) genetic background. Diagrammatic representation of overall backcrossing scheme (A). Schematic representation of experimental aim and summary of nomenclature used (B). Genome of the MHco4(WRS) ivermectin resistant strain represented in red and the genome of the MHco3(ISE) susceptible reference strain represented in blue.

The artificial selection of resistance by serial passage and underdosing of susceptible laboratory strains has been undertaken by a number of groups in the past ([23] in sheep, [24] in rodents and [25]–[28] *in vitro*). However, a major limitation of such approaches is that selection in the real world is very different to that applied in the laboratory [20]. A more powerful approach is to take strains of parasites in which resistance was originally selected in the field and genetically map the anthelmintic resistance loci. To undertake detailed genetic mapping, a number of things are necessary. Firstly, the ability to undertake genetic crossing in the organism. Secondly, to have characterised genetically distinct (preferably isogenic) resistant and susceptible isolates on which to undertake mapping crosses. Thirdly, a fully sequenced and assembled genome (or at least a detailed genetic map) for the organism. All of these are achievable for *H. contortus* making genetic mapping in this organism a feasible objective in the future [14]. However, a number of other genetic strategies which, although short of classical genetic mapping, can potentially improve our ability to use genome-wide approaches for the identification of anthelmitic resistance genes in the short term. One example of such an approach is the introgression of resistance genes from field derived strains into a characterized susceptible genetic background with repeated backcrossing. This would allow whole genome or candidate gene comparisons such as transcriptomics and genome-wide polymorphism analysis to be more meaningfully applied and interpreted since differences between backcrossed resistant strains and the susceptible parental isolate would be limited to those regions of the genome linked to resistance-conferring loci (Figure 1A).

In this paper we report the introgression of ivermectin resistance-conferring loci from two different ivermectin resistant strains, into the genetic background of the susceptible genome reference strain MHco3(ISE) [29]. We have used microsatellite markers to monitor the backcrossing and to genetically validate the success of the approach. We also have preliminary evidence of potential linkage of one marker to a resistance conferring locus. This work provides an important proof of concept of this novel genetic approach for parasites and has generated powerful tools to investigate the genetic basis of ivermectin resistance.

## Materials and Methods

### Ethics statement

All experimental procedures described in this manuscript were examined and approved by the Moredun Research Institute Experiments and Ethics Committee and were conducted under approved British Home Office licenses in accordance with the Animals (Scientific Procedures) Act of 1986. The Home Office licence number is PPL 60/03899 and experimental IDs for these studies were E06/58, E06/75 and E09/36.

### *H. contortus* strains

The MHco3(ISE) strain [29] is the product of multiple rounds of inbreeding, is susceptible to all main classes of anthelmintics and has been adopted as the standard genome strain for the *H. contortus* sequencing project at the Wellcome Trust Sanger Institute (http://www.sanger.ac.uk/Projects/H_contortus/). In spite of its inbreeding history it retains high levels of genetic polymorphism [10], [21]. The White River [30] and Chiswick avermectin resistant [31] strains of *H. contortus*, were originally isolated from South Africa and Australia respectively. Subsequently they have been experimentally passaged through sheep for a number of years at the Moredun Research Institute, and these versions of the strains are designated as MHco4(WRS) and MHco10(CAVR) respectively throughout the manuscript. These strains were chosen for this work for several reasons: Firstly, their origins were from the field and have subsequently been well characterized in the laboratory [10], [21], [30], [31] Secondly, we have previously shown that these are genetically divergent with respect to the susceptible MHco3(ISE) strain. This is important as it allows us to distinguish between resistance and susceptible parental genotypes when genetic markers are analyzed in the backcross progeny. Thirdly, they were originally derived from different continents –MHco4(WRS) from Africa and MHco10(CAVR) from Australia- and so it is highly likely that ivermectin resistance has been independently selected in each strain. This allows a potentially interesting comparison of resistance mechanisms from two independently selected strains.

### Backcrossing scheme and nomenclature

The basic approach was to cross male worms of the MHco3(ISE) susceptible strain with female worms from the resistant strains in the initial cross. Subsequently F_1_ female worms derived from each generation of backcross were crossed again with male MHco3(ISE) worms (Figure 1B). The F_1_ progeny of the first genetic crosses between MHco3(ISE) and the two ivermectin resistant strains MHco4(WRS) and MHco10(CAVR) were designated as MHco3/4 and MHco3/10 respectively. The nomenclature for subsequent backcrosses was MHco3/4.BC_n_ and MHco3/10.BC_n_, denoting the Moredun Research Institute (M), *H. contortus* (Hco), the unique numbers allocated to both parental strains (3/4 or 3/10), and the backcross generation (BC_2, 3 and 4_) (Figure 1). Progeny of each cross were collected and cultured to L_3_ in the standard way. They were then used to infect donor sheep to generate L_4_ worms for the next backcross. These donor sheep were treated with ivermectin to ensure only worms which were phenotypically ivermectin resistant were used in the next backcross (see following section for details). The passage of these larvae through three more rounds of ivermectin selection and crossing against the ivermectin susceptible isolate (MHco3(ISE)) produced a final fourth backcross generation (MHco3/4.BC_4_ or MHco3/10.BC_4_) (Figure 1B).

### Harvesting and preparation of L4 worms for surgical transplantation

Crosses between two strains were performed by surgically transplanting approximately 50 late L_4_ male worms from one strain and 50 late L_4_/adult female worms from the other strain directly into the abomasum of a recipient sheep. In order to produce L_4_ for transplantation male worm-free donor lambs were orally dosed with between 5,000–10,000 L_3_ of either MHco3(ISE), or the ivermectin resistant strain to be crossed against. *H. contortus* donors with the ivermectin resistant strains were treated with 0.1 mg/kg of ivermectin (Oramec drench for sheep; Merial) on day 10 or 11 post infection to select for ivermectin resistant progeny prior to transplantation. Donor sheep were euthanased on day 14 post infection and worms were harvested from their abomasa (day 14 female worms of these strains have previously been shown to be sexually immature and not produce viable progeny; [32]). The abomasal contents and washings containing the nematodes were first passed through a 1 mm sieve, transferred into fresh physiological saline (0.85% NaCl) and then maintained at 37°C. Male and female *H. contortus* could then be picked into pre-warmed petri-dishes containing RPMI 1640 tissue culture medium in readiness for surgical transfer into the abomasa of worm-free recipient sheep. 45–100 male late L_4_/immature adult MHco3(ISE) *H. contortus* and 50–100 female late L_4_/immature adult *H. contortus* were surgically transferred into the abomasa of male worm free recipient lambs, within 2 hours of recovery from the donor sheep.

### Setting up genetic crosses by surgical transplantation

Recipient sheep were anaesthetised to allow a 10 cm vertical incision to be made through the skin, underlying fascia, muscle and peritoneum, over the right flank, midway between the last rib and pelvis and about 10 cm above the midline. The abomasum was located and partially exteriorised, to enable a 1 cm diameter sub-serosal purse-string suture to be placed. A stab incision was then made in the centre of the purse-string suture, through which 50 male late L_4_/immature adult MHco3(ISE) *H. contortus* and 50 female late L_4_/immature adult MHco4(WRS), or MHco10(CAVR) *H. contortus* (or subsequent backcross generations) were introduced into the abomasum in approx 5 ml RPMI, using a 5 mm diameter blunt ended, glass pipette. The purse-string suture was then closed and the surgical incision repaired allowing the completion of surgical transfers within about 2 hours from the recovery of the nematodes from the donor sheep. Sheep were routinely injected with 1 mg/kg meloxicam (Metacam 20 mg/ml solution for injection; Boehringer Ingelheim) for post-surgical analgesia and 7 mg/kg amoxicillin/1.75 mg/kg clavulanic acid (Synulox ready-to-use injection; Pfizer) and closely monitored on completion of the surgery. No adverse effects were noted during the course of this study. Eggs were identified in the faeces approx 3 days post surgery and collected daily and coprocultured to produce L_3_.

### Controlled efficacy test to determine ivermectin sensitivity of backcross strains

A controlled efficacy test (CET) using ivermectin was undertaken on the two separate fourth generation backcross strains, MHco3/4.BC_4_, and MHco3/10.BC_4_ alongside the three original parental strains used in this study, namely MHco3(ISE), MHco4(WRS) and MHco10(CAVR). Seventy-five parasite naïve lambs were divided equally between the five different strains. For each strain, the 15 lambs were further allocated into three treatment groups of five animals: no treatment, ivermectin (0.2 mg/kg BW) or ivermectin (0.1 mg/kg BW). Lambs were initially allocated randomly to strain and treatment groupings that were subsequently balanced, where needed on the basis of sex and the weight of the animal just prior to the experiment, to ensure that groups were as similar as possible. The lambs were infected with 5,000 *H. contortus* L_3_ on day 0. Faecal worm egg counts (FEC) were conducted [33] at the start of the controlled efficacy test to confirm their parasitic nematode-free status and on days 16, 18, 21, 28, 29 and 36 pi to monitor the counts. On day 29 post infection (pi) the lambs were weighed again and orally dosed where appropriate with the correct volume of ivermectin (Oramec drench for sheep; Merial) using a syringe [34]. All of the lambs were euthanased on day 36pi and worms harvested from their abomasa for determination of *H. contortus* burdens in abomasal saline washings and digests [34]–[35]. *H. contortus* recovered from 2% (MHco4 and MHco10) and 10% (MHco3, MHco3/4.BC_4_ and MHco3/10.BC_4_) sub-samples of the abomasal washings and digests were counted and sexed (only adults were seen), the higher sub-sample volume was examined in the backcross strains due to the smaller numbers of worms present. The percentage efficacies of each anthelmintic treatment were calculated using the equation 100 (1−T/C), where T and C are the arithmetic mean total *H. contortus* burdens of the treated and control groups respectively [36]. The same equation 100 (1−T/C) was also used for the calculation of percentage treatment efficacy using faecal egg counts of treated and control groups that were taken 7 days post treatment, on day 36pi at necropsy. For all estimates of efficacy 95% confidence intervals (CIs) were calculated [36] and anthelmintic resistance was deemed to be present when the percentage efficacy of reduction of parasitic nematode burdens or FECs was less than 95% [37]. In addition, the mean treatment and upper and lower 95% confidence intervals were calculated on the FEC data using Bootstrap analysis and a resampling number of 2000 using the “BootStreat” program [38] cited in [39].

### Molecular genotyping and genetic analysis

All microsatellite genotyping, on both bulk and single worm DNA lysates, was performed using the same PCR amplification methods and parameters previously described [21]. Capillary electrophoresis was performed using an ABI Prism 3100 genetic analyzer (Applied Biosystems, Foster City, CA) for the accurate sizing of microsatellite PCR products. The forward primer of each microsatellite primer pair was 5′-end labeled with FAM, HEX, or NED fluorescent dyes (MWG) and electrophoresed with GeneScan ROX 400 (Applied Biosystems) internal size standard. Individual chromatograms were analyzed using Genemapper Software Version 4.0 (Applied Biosystems).

Bulk worm DNA lysates were made as previously described [21]. Duplicate bulk lysates were made using approximately 500 L_3_ worms from each generation of the backcrossing procedure (BC_1_, BC_2_, BC_3_, BC_4_), the F_1_ progeny of the initial genetic crosses (MHco3/4 and MHco3/10) and L_3_ from the three parental strains MHco3(ISE), MHco4(WRS) and MHco10(CAVR).

In addition to the bulk worm DNA preparations, 30–40 individual (L_3_ or adult) worm DNA lysates were prepared from seven strains for more detailed genetic analysis: the three parental strains MHco3(ISE), MHco4(WRS) and MHco10(CAVR); the two backcross strains, MHco3/4.BC_4_ and MHco3/10.BC_4_ and the two populations of survivors (0.1 mg/kg ivermectin) of both the backcross strains (taken from appropriate control and the ivermectin treated animals from the controlled efficacy test experiment respectively).

The genotyping of parasite populations/strains by amplifying a microsatellite from “bulk” DNA lysates made from a population of worms has been previously described [21]. It is a valuable approach to quickly “fingerprint” worm populations for the presence or absence of microsatellite alleles and gives an approximation as to their relative frequencies. 18 microsatellite loci were used for genotyping the bulk worm DNA lysates. These included 13 previously well characterised loci: Hcms25, Hcms27, Hcms33, Hcms36, Hcms40 [40]; Hcms8a20, Hcms22co3 [21]; HcmsX142, HcmsX146, HcmsX151, HcmsX182, HcmsX256, HcmsX337 [10] and five new loci Hcms3561, Hcms18210, Hcms26981, Hcms40506, Hcms18188 (Supplementary Table S1). Nine microsatellite loci, chosen for their ability to differentiate between the three parental strains, were used to genotype the individual worm lysates from the seven key *H. contortus* strains. These were Hcms27, Hcms36, Hcms40, Hcms8a20, Hcms22c03 and four recently identified loci, namely, Hcms3086, Hcms22193, Hcms44104 and Hcms53265 (Redman *et al.*, in preparation).

For the single worm genotype data, Pairwise F_ST_ values were calculated using Arlequin version 3.11 [41]. Data were defined as “standard” rather than microsatellite, as it did not necessarily adhere to stepwise mutation model. PCA was performed using GenAlEx version 6 [42] preserving individual worm genotypes.

## Results

### Phenotypic analysis of parental and backcross populations

**Figure 2:**
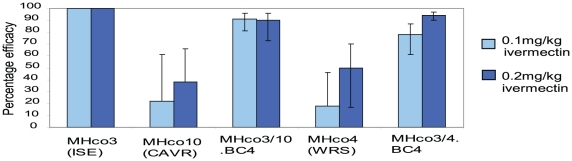
Efficacy of ivermectin against *H. contortus* parental and backcross strains resulting from controlled efficacy experiment. Percentage efficacies of ivermectin at two different therapeutic doses (0.1 mg/kg and 0.2 mg/kg) against the three parental strains and the backcross strains, estimated by comparison of mean worm burden of treatment and control group. Y-error bars represent 95% confidence intervals.

The percentage efficacy of ivermectin at doses 0.1 mg/kg and 0.2 mg/kg was determined from *H. contortus* arithmetic mean burdens of treated and control groups (Supplementary Figure S1). Ivermectin efficacies were 100, 91, 78, 22 and 18% at 0.1 mg/kg and 100, 90, 94, 38 and 50% at 0.2 mg/kg against the MHco3(ISE), MHco3/10.BC_4_, MHco3/4.BC_4_, MHco10(CAVR) and MHco4(WRS) strains respectively (Figure 2 and Supplementary Table S2). Hence, the resistance phenotypes of the parental strains MHco4(WRS) and MHco10(CAVR) was confirmed as was the presence of resistant parasites in the backcross populations MHco3/10.BC_4_, MHco3/4.BC_4_.

Treatment efficacies based on faecal egg count reduction for the MHco3(ISE), MHco3/10.BC_4_, MHco3/4.BC_4_, MHco10(CAVR) and MHco4(WRS) strains were 100, 89, 69, 41 and 0% at 0.1 mg/kg IVM and 100, 92, 93, 37 and 36% at 0.2 mg/kg IVM respectively when compared to untreated controls (Supplementary Table S3).

### Monitoring the backcrossing procedure by microsatellite genotyping of bulk worm DNA preparations

The genotyping of parasite populations/strains by amplifying a microsatellite from “bulk” DNA lysates made from a population of worms has been previously described [21]. Although, this technique cannot give accurate allele frequency data for alleles present at low frequency in the parasite population, it is a rapid approach to “fingerprint” worm populations for the presence or absence of microsatellite alleles and to obtain approximate frequencies for the predominant alleles. Consequently, we used this as a means of monitoring the backcrossing procedure as it progressed. The alleles from all 18 microsatellite loci were scored as either being ‘present’ or ‘absent’ in genotypes derived from the bulk DNA lysate preparations of the parental isolate, the F_1_ strains and each backcross generation (Supplementary Table S4 and S5). When the parental strains MHco3(ISE) and MHco4(WRS) are compared, a total of 17 different isolate-specific alleles were identified across 9 different loci: 5 alleles present in MHco3(ISE) but absent from MHco4(WRS) and 12 alleles present in MHco4(WRS) but absent from MHco3(ISE). Similarly, comparison of the parental strains MHco3(ISE) and MHco10(CAVR) revealed a total of 41 isolate-specific alleles across 16 loci: 19 alleles present in MHco3(ISE) but absent from MHco10(CAVR) and 22 alleles present in MHco10(CAVR) but absent from MHco3(ISE). This is consistent with our previous report that both ivermectin resistant strains are genetically distinct from the MHco3(ISE) susceptible reference strain with MHco10(CAVR) being more genetically divergent than MHco4(WRS) [10], [21]. For both backcrosses, almost all MHco3(ISE) specific alleles were maintained through the 4 backcross generations and were still present in the MHco3/4BC_4_ and MHco3/10BC_4_ strains. The only exceptions were the Hcms25, 215 bp and 217 bp alleles which were present in the first backcross strain (MHco3/10.BC_1_) but were lost at the second backcross generation (MHco3/10.BC_2_). These were relatively rare alleles in MHco3(ISE) strain - allele 215 bp present at 3.6% and allele 217 bp present at 11.1% - and therefore their loss could be due to purely stochastic reasons. In contrast, almost all alleles specific to the two ivermectin-resistant strains MHco4(WRS) or MHc010(CAVR), disappear during the backcross procedure and are absent in the MHco3/4BC_4_ and MHco3/10BC_4_ strains. There are only two exceptions to this. First, the HcX256 allele 243 bp, which is retained in MHco3/10BC_4_. However, it was only detected at a frequency of 4.6% in MHco3/10BC_4_ compared with its original frequency of 38% in the MHco10(CAVR) parental strain and so although not completely eliminated, this allele has undergone a dramatic reduction in frequency during the backcrossing procedure (Supplementary Figure S2). The second exception is the MHco10(CAVR)-specific alleles, 244 bp and 248 bp and the MHco4(WRS)-specific allele 244 bp of loci Hcms8a20. These are maintained throughout all generations of both backcrosses and this is presented in more detail in the following sections. However, overall the MHco3/4.BC_4_ and MHco3/10BC_4_ strains have a similar genetic background to the MHco3(ISE) parental strain based on bulk genotyping with a panel of 18 microsatellite loci as would be predicted from the backcrossing scheme.

### Genetic analysis by individual genotyping of parental and backcrossed strains

**Figure 3:**
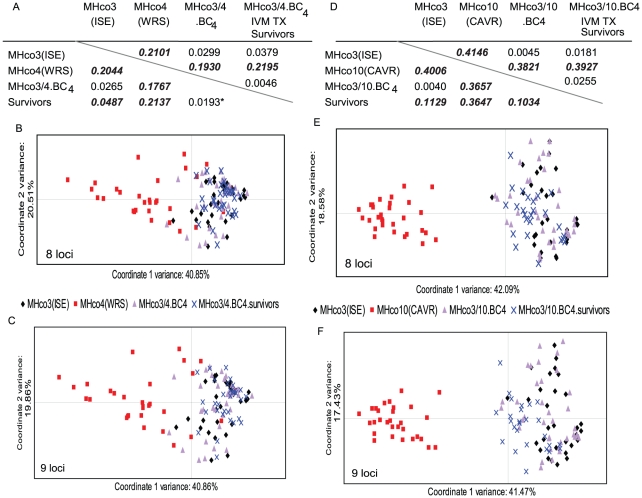
Visualisation of genetic differentiation between *H. contortus* parental and backcross strains. Pairwise F_ST_ estimates and principal component analysis of parental and 4^th^ generation backcross isolates pre and post ivermectin treatment (0.1 mg/kg ivermectin) using multi-locus genotype data. Figure 3A, B and C shows the data for the MHco3/4 cross and Figure 3D, E and F for the MHco3/10 cross. Both F_ST_ and PCA analysis was performed with 9 loci and also with 8 loci (when Hcms8a20 was excluded from this latter analysis, since it shows evidence of genetic linkage to the ivermectin resistance phenotype). Figure A and D show pairwise F_ST_ estimates: values in the tables below the diagonal are for analysis of all 9 loci and above the diagonal for the 8 loci (excluding Hcms8a20). Genetic differentiation between isolates at significance level, p<0.01 (highlighted in bold and italics). Asterisk indicates level of genetic differentiation is marginal (significance at p<0.05). PCA analysis for all 8 loci are shown in panels B and E and for the 9 loci (including 8a20) in panels C and F. Each data point represents a single worm based on a multi-locus genotype of 9 or 8 markers.

The parental strains and final backcross populations (MHco3/4.BC_4_ and MHco3/10.BC_4_) were analysed in more detail by genotyping 30–40 individual worms with 9 of the most discriminatory microsatellite markers (Figure 3). The data is presented separately with marker Hcms8a20 either excluded (8 loci data) or included (9 loci data) since this marker shows evidence of an association with the ivermectin resistance phenotype (see next section). Pairwise F_ST_ estimates based on the multi-locus genotype data revealed a high level of genetic differentiation between the parental strains: MHco3(ISE) and MHco4(WRS) had a high level of genetic differentiation (8 loci F_ST_ = 0.2101, 9 loci F_ST_ = 0.2044, Figure 3A) and MHco3(ISE) and MHco10(CAVR) an even higher level (8 loci F_ST_ = 0.4146, 9 loci F_ST_ = 0.4006, Figure 3D). Hence MHco10(CAVR) is slightly more divergent from MHco3(ISE) than is MHco4(WRS) confirming previous comparative analysis of these strains [21]. This genetic differentiation between the parental strains was also demonstrated by principal component analysis of individual worm multi-locus genotypes (Figure 3B, C, E and F). Both of the ivermectin resistant strains ((MHco4(WRS) and MHco10(CAVR)) form clusters distinct from the MHco3(ISE) cluster.

Both the BC_4_ backcross strains show a very low level of genetic differentiation from the MHco3(ISE) parental strain (8 loci F_ST_ = 0.0299 and 9 loci F_ST_ = 0.0265 for MHco3/4.BC_4_ and 8 loci F_ST_ = 0.0045 and 9 loci F_ST_ 0.0040 for MHco3/10.BC_4_). In contrast they show a high level of genetic differentiation from the ivermectin resistant parental isolates (8 loci F_ST_ = 0.1930 and 9 loci F_ST_ = 0.1767 between MHco4(WRS) and MHco3/4.BC_4_ (Figure 3A) and 8 loci F_ST_ of 0.3821 and 9 loci F_ST_ of 0.3657 between MHco10(CAVR) and MHco3/10.BC_4_ (Figure 3D)). Indeed, this level of genetic differentiation is of a similar magnitude as that seen between the original parental strain of each cross. These results are again supported by the principal component analysis of multi-locus genotypes of single worms where both the MHco3/4.BC_4_ and MHco3/10.BC_4_ populations are distinct from the MHco4(WRS) and MHco10(CAVR) strains respectively and cluster with the MHco3(ISE) strain (Figure 3B, C, E and F). This demonstrates the genetic background of the 4^th^ generation backcross strains is similar to that of the MHco3(ISE) parental strain.

### Genetic analysis of worms from MHco3/4.BC_4_ and MHco3/10.BC_4_ backcross strains that are phenotypically resistant to ivermectin

Although the MHco3/4.BC_4_ and MHco3/10.BC_4_ backcross populations are ivermectin resistant based on the CET, they are significantly less resistant than the original MHco4(WRS) and MHco10(CAVR) parental strains. This is not unexpected given the nature of the backcrossing scheme (see discussion below) and means the backcross strains consist of a mixed population of worms of differing ivermectin resistant phenotypes. In order to genetically characterize those worms that were phenotypically resistant to ivermectin at a dose which is 100% effective for the MHco3(ISE) isolate, we infected two sheep each with MHco3/4.BC_4_ and MHco3/10.BC_4_, treated with 0.1 mg/kg and harvested and prepared DNA from adult worms that survived this drug treatment. These worms surviving ivermectin treatment were then individually genotyped with the 9 microsatellite markers and F_ST_ and PCA analysis was performed initially with 8 loci (loci Hcms8a20 excluded) and subsequently with loci Hcms8a20 included in the analysis (9 loci) (Figure 3). On the basis of the eight markers, the ivermectin resistant individuals within the MHco3/4 backcross strain were genetically very closely related to the parental susceptible MHco3(ISE) strain, (8 loci F_ST_ = 0.0379) and genetically divergent from the ivermectin resistant parental strain (8 loci F_ST_ of 0.2195 for MHco4(WRS)) (Figure 3A). Similarly, the phenotypically ivermectin resistant worms within the MHco3/10.BC_4_ were more genetically similar to the MHco3(ISE) parental strain (8 loci F_ST_ = 0.0181) than to the MHco10(CAVR) parental strain (8 loci F_ST_ = 0.3927) (Figure 3D).

Inclusion of the Hcms8a20 locus into the analysis produced a similar result but slightly reduced the overall genetic differentiation between the ivermectin resistant backcross worms from each of the resistant parental strains (9 loci F_ST_ = 0.2137 vs 8 loci F_ST_ = 0.2195 between the MHco3/4.BC_4_ ivermectin survivors and MHco4(WRS) strain and 9 loci F_ST_ = 0.3647 vs 8 loci F_ST_ = 0.3927 between MHco3/10.BC_4_ ivermectin survivors and MHco10(CAVR) (Figure 3A and D)). Conversely, inclusion of the Hcms8a20 locus increases the genetic differentiation between the ivermectin resistant backcross survivors and the susceptible MHco3(ISE) parental strain to a point where it is statistically significant: 9 loci F_ST_ = 0.0487 vs 8 loci F_ST_ = 0.0379 between the MHco3/4.BC_4_ ivermectin survivors and MHco3(ISE) and 9 loci F_ST_ = 0.1129 vs 8 loci F_ST_ = 0.0181 between the MHco3/10.BC_4_ ivermectin survivors and MHco3(ISE) (Figure 3A and D; statistically significant genetic differentiation between any pair of strains highlighted in italics).

### Evidence of linkage of marker Hcms8a20 to an ivermectin resistance-conferring locus

**Figure 4:**
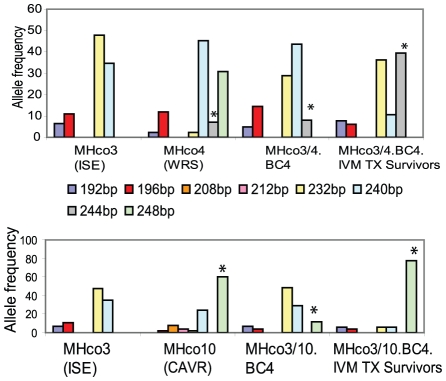
Allele frequencies of locus Hcms8a20 for *H. contortus* parental and backcross strains. Allele frequencies based on genotyping individual worms (parental strains, n = 30; the 4^th^ generation backcross strains: MHco3/4.BC_4_, n = 35 and MHco3/10.BC_4_, n = 38; and 4^th^ backcross generation survivors of ivermectin treatment at 0.1 mg/kg ivermectin: MHco3/4.BC_4_.survivors, n = 33 and MHco3/10.BC_4_.survivors, n = 26). Asterisk indicates MHco4(WRS) and MHco10(CAVR)-specific alleles retained in the backcross strains.

Consistent with the PCA and F_ST_ analysis, examination of the allele frequency data derived from the single worm genotyping revealed that for eight of the nine markers, the allele frequency histograms of the ivermectin treatment survivors of strains MHco3/4.BC_4_ and MHco3/10.BC_4_ were very similar to the MHco3(ISE) susceptible parental strain and divergent from the resistant parental strains MHc04(WRS) and MHco10(CAVR) respectively (see Supplementary Figure S3A–H). However, this was not the case for Hcms8a20. In this case, for both backcross strains, an allele that was frequent in the resistant parental strain was retained in the fourth generation backcross strains (Supplementary Table S4, S5 and Figure 4). In the case of the MHco3/4 backcross, allele 244 bp (which was specific to the MHco4(WRS) parent) was retained at a frequency of 8% in the MHc3/4.BC_4_ worms. Notably this increased to a frequency of 40% in the population of MHc3/4.BC_4_ worms that survived 0.1 mg.kg ivermectin treatment (Figure 4). Similarly, for the MHco3/10 backcross, allele 248 bp (which was specific to the MHco10(CAVR) parent) was retained at a frequency of 12% in MHco3/10.BC_4_ worms. Again, this increased to a frequency of 78% in the MHco3/10.BC_4_ worms that survived 0.1 mg/kg ivermectin treatment (Figure 4).

## Discussion

### Introgression of ivermectin resistance genes into the MHco3(ISE) susceptible genome reference strain

*H. contortus* is one of the few parasitic nematodes where genetic crosses are currently possible. Previous genetic crossing experiments have been performed to assess the level of dominance of resistance genes and test for evidence of linkage of a P-glycoprotein with the ivermectin resistance phenotype [9], [11], [43]–[45]. More recently, a genetic mapping approach was undertaken in which resistant F_2_ were selected and AFLP used to look for markers associated with resistance [8]. This was a potentially powerful approach although, in that case, the ability to analyze the F_2_ progeny was limited by the lack of genetic differentiation of the parental strains used. We have taken a different genetic approach in which we have successfully introgressed regions of the *H. contortus* genome containing loci conferring ivermectin resistance from two different ivermectin resistant strains into the genetic background of the MHco3(ISE) susceptible strain. This latter strain is susceptible to the main classes of anthelmintics and is currently being used as the reference strain for the *H. contortus* genome sequencing project (http://www.sanger.ac.uk/resources/downloads/helminths/haemonchus-contortus.html). The introgression of resistance genes into this strain was achieved by repeated backcrossing of the MHco4(WRS) and MHco10(CAVR) strains against the MHco3(ISE) strain with the application of ivermectin selection at each backcross. A therapeutic dose of 0.1 mg/kg ivermectin was chosen as an appropriate discriminatory dose for selection because it is 100% effective against the parental MHco3(ISE) strain (F. Jackson, unpublished data). This was confirmed by our controlled efficacy test; not a single worm of the MHco3(ISE) strain could be found surviving treatment at this dose rate in any of the five treated sheep (Supplementary Figure S1). In contrast, for both the backcross isolates MHco3/4.BC_4_ and MHco3/10.BC_4_, a proportion of worms survived ivermectin treatment at dose both the 0.1 and 0.2 mg/kg BW dose rates demonstrating these surviving individuals were phenotypically resistant to ivermectin.

Although both backcross strains contained individuals that were phenotypically resistant to ivermectin treatment at doses 0.1 and 0.2 mg/kg, the overall resistance level of the backcross strains was significantly lower than either of the parental resistant strains. This is unsurprising given the nature of the backcrossing regime and experimental design: In order to produce enough infective larvae to undertake a controlled efficacy test, the F_1_ progeny of the fourth backcross were used to infect a donor sheep which was not treated with drug. Since any resistance alleles would be heterozygous in the F_1_ of the fourth backcross, resistance alleles would segregate during sexual reproduction of the worms in the final donor sheep that was used to produce L_3_ for the CET. Hence, the final backcross populations used in the CET would consist of a mixture of resistant and susceptible worms. The relatively low proportion of individual backcrossed worms with an ivermectin resistant phenotype in the CET is consistent with the hypothesis that multiple additive loci contribute to ivermectin resistance since only those worms in which several resistance loci have segregated would be resistant to the doses of drug used. Of course, different alleles can differ in their magnitude of effect, their level of dominance and their expressivity and so the overall relationship between genotype and phenotype is potentially complex.

The important point is that backcross strains contain a proportion of individuals that are phenotypically resistant to ivermectin (unlike the MHco3(ISE) parental susceptible strain). The observation that some worms in the backcross strains survive treatment at this dose demonstrates that the resistance-conferring alleles have been successfully introgressed from the parental ivermectin resistant isolates MHco4(WRS) and MHco10(CAVR). Importantly, when individual worms from the MHco3/4.BC_4_ and MHco3/10.BC_4_ backcross strains that survive the 0.1 mg/kg ivermectin treatment were genotyped with our microsatellite markers, their genetic background was very similar to that of the susceptible MHco3(ISE) parental strain and highly differentiated from the MHco4(WRS) and MHco10(CAVR) resistant parental strains (Figure 3). This demonstrates that these individuals contain resistance-conferring loci, derived from the resistant parental strains, but have a MHco3(ISE) susceptible genetic background across most of the genome. Hence these strains now provide a powerful resource on which to apply functional genomic strategies to identify regions of the genome harbouring resistance loci. Comparative analysis of ivermectin resistant individuals from the MHco3/4.BC_4_ and MHco3/10.BC_4_ backcross strains with the parental isolates can be undertaken to identify regions of the genome derived from the MHco10(CAVR) and MHco4(WRS) parental strains and hence harbouring resistance conferring loci. Such analyses could include genome-wide polymorphism analysis, RNAseq analysis (to examine expression profiles and coding-region polymorphisms) or targeted analysis of candidate genes. It is important to note that it is likely that the introgressed regions of the MHco4(WRS) and MHco10(CAVR) are relatively large since just four generations of backcrossing have been performed and recombination will have had limited opportunity to break down genetic linkage. Nevertheless analysis of these strains should provide the locations of major ivermectin resistance loci in the *H. contortus* genome. Further backcrossing, together with improving genomic resources for this parasite, will provide the opportunity to iteratively interrogate these strains to identify the genomic location of resistance loci more accurately.

### Evidence for genetic linkage of marker Hcms8a20 to a resistance conferring loci

Of the 18 microsatellite markers that were used to monitor the backcrossing procedure, based on the presence and absence of strain-specific alleles, there was only one in which alleles specific to the parental resistance strains were retained in the 4^th^ generation backcross progeny for both back crosses. This was marker Hcms8a20. Furthermore, when the F_ST_ and PCA analysis were performed using the nine most discriminatory markers, each single marker was iteratively excluded to check for distortions of the data due to any effects of single markers. The only marker whose exclusion had any discordant effect on the data was Hcms8a20 (Data not shown). The exclusion of the loci Hcms8a20 from the F_ST_ and PCA analysis revealed that MHco3/4.BC_4_ and MHco3/10.BC_4_ ivermectin resistant worms (survivors), the susceptible MHco3(ISE) strain and their respective backcross strains were all genetically indistinguishable. The inclusion of the loci Hcms8a20 into the same analysis increased the level of genetic differentiation between these aforementioned strains of worms to the point of statistical significance. Examination of the individual allele frequencies for this marker confirms that the allelic profile was more similar to the resistant parental strains than the MHco3(ISE) parental strain (Figure 4). Indeed, a single allele, specific to the respective resistant parental strains was retained in the two backcross populations. These are present at relatively low frequency (8% for allele 244 bp in MHc3/4 BC_4_ and 12% for allele 248 bp in MHc3/10.B_4_). However, these are present at much higher frequencies in the populations of backcross worms that survive 0.1 mg/ml ivermectin treatment (40% for allele 244 bp in MHc3/4 BC_4_ and 78% for allele 248 bp in MHc3/10.B_4_). It is impossible to predict the precise changes in allele frequency one would expect at a single locus during the backcrossing procedure, or following drug selection, when several loci may have differing additive contributions to the overall resistance phenotype. However, the fact that the same locus, Hcms8a20, shows evidence of retention of alleles specific for the parental resistant isolates in both fourth generation backcross strains, together with the dramatic increase in frequency of these in the phenotypically ivermectin resistant worms (relative to the unselected backcross populations) provides strong evidence that this locus is linked to a resistance conferring polymorphism. The fact that different alleles appear to be selected from the two different parental resistant strains is not necessarily surprising. These two strains – MHco4(WRS) and MHco10(CAVR) - are genetically divergent and originally derived from disparate geographical regions. Consequently, it is entirely possible that a resistance-conferring polymorphism would be genetically linked to different haplotypes of adjacent markers. As the *H. contortus* genome project progresses it will be interesting to “walk out” from the Hcms8a20 marker to examine additional linked markers to define the size of the region showing evidence of linkage disequilibrium. Furthermore, we hypothesize that additional loci contribute to the ivermectin resistant phenotype of the MHco4(WRS) and MHco10(CAVR) parental strains. We anticipate that these may be identified as we iteratively interrogate the backcross strains with larger marker panels as they become available form the *H. contortus* genome sequencing project. Similarly, the backcross strains now represent a powerful genetic resource with which to determine if the various candidate genes identified from other studies contribute to the ivermectin resistance phenotype of the MHco4(WRS) or the MHco10(CAVR) strains.

In summary, we describe the introgression of resistance-conferring loci from two independent ivermectin resistant strains into a susceptible reference strain of *H. contortus*. This is a novel approach that provides a powerful adjunct to both candidate gene and whole genome analysis aimed at identifying anthelmintic drug resistance loci. The continued advancement of such genetic approaches, alongside genomic resources for *H. contortus*, should allow this organisms to be used in an increasingly powerful manner to study the genetic basis of anthelmintic resistance in strongylid nematode parasites.

## Supporting Information

Figure S1 **Total worm burden of lambs.** Total worm burden of lambs following therapeutic dose (0.1 mg/kg and 0.2 mg/kg) of ivermectin against the three parental isolates and the backcross isolates. Mean worm burden per treatment group indicated by trend lines.
(EPS)

Figure S2 **Allele frequency histograms of loci HcmsX256 for** ***H. contortus*** **parental and backcross strains.** Allele frequencies resulting from individual worm genotyping of populations of 30 single worms confirm the presence of the MHco10(CAVR)-specific allele, 243 bp at the very low level of 4.6% in the MHco3/10.BC_4_ strain.
(EPS)

Figure S3 **Allele frequency histograms of eight loci for** ***H. contortus*** **parental and backcross strains.** Allele frequencies of the microsatellite loci, Hcms27 (A), Hcms36 (B), Hcms40 (C), Hc53265 (D), Hcmc22c03 (E), Hc22193 (F), Hcms3086A (G) and Hc44104 (H) for parental strains (based on genotyping 30 individual worms per strain i.e. n = 30), the two 4^th^ generation backcross strains (MHco3/4.BC_4_, n = 35 and MHco3/10.BC_4_, n = 38) and 4^th^ backcross generation survivors of ivermectin treatment (0.1 mg/kg ivermectin) (MHco3/4.BC_4_.survivors, n = 33 and MHco3/10.BC_4_.survivors, n = 26).
(EPS)

Table S1 **Summary information for the five new** ***H. contortus*** **microsatellite markers.** Sequence of repeat and primers used to amplify microsatellite loci.
(DOC)

Table S2 **Treatment efficacies based on worm burden.** Arithmetic mean (±SEM) and range of *H. contortus* counts, sex differentiation of worm burdens and percentage efficacies.
(DOC)

Table S3 **Treatment efficacies based on faecal egg count reduction.** Arithmetic mean (±SEM) and range of faecal egg count and percentage efficacy seven days post-treatment.
(DOC)

Table S4 **Genetic profiles of backcrossed strains derived from MHco4(WRS) monitored by bulk worm microsatellite “finger-printing”.** Alleles present in the bulk worm preparations of the parental (MHco3(ISE) and MHco4(WRS)), F_1_ and backcross strains.
(XLS)

Table S5 **Genetic profiles of backcrossed strains derived from MHco10(CAVR) monitored by bulk worm microsatellite “finger-printing”.** Alleles present in the bulk worm preparations of the parental (MHco3(ISE) and MHco10(CAVR)), F_1_ and backcross strains.
(XLS)
