# Supplementary material for: Introgression of Ivermectin Resistance Genes into a Susceptible Haemonchus contortus Strain by Multiple Backcrossing
Source: PLoS Pathog. 2012 Feb 16;8(2):e1002534. doi: 10.1371/journal.ppat.1002534 (PMC3280990; doi:10.1371/journal.ppat.1002534)
Supplement: Table S1 — Summary information for the five new H. contortus microsatellite markers. Sequence of repeat and primers used to amplify microsatellite loci. (DOC) [file ppat.1002534.s004.doc]

**Table S1. Summary information for the five new *H. contortus* microsatellite markers. Sequence of repeat and primers used to amplify microsatellite loci.**

| Loci | Repeat sequence | Primer sequences (5’ → 3’) |
| --- | --- | --- |
| Hc3561 | (GTT)_6_ GTCTTT (GTT)_2_ | F: CCTACATGTCTCCCATATGTC, R: TTAGCGAAGTAATAGCGTGCC |
| Hc18210 | TGC TC (TGC)_9_ | F: TCAGGAGTTCGGCTTTTCAG, R: GTGAGCTGAGCTTCGTTAGAG |
| Hc26981 | (CATAAC)_4_ AATAC CATAAC | F: ACACTCTTATCACGCTACCTG, R: CTCACTTTCCCAGTCTTATCG |
| Hc40506 | (GTCT)_2_ CTCT (GTCT)_4_ GTAT (GTCT)_20_ (GTAT)_3_ (GTCT)_9_ GCCT (GTCT)_6_ GTCC | F: CGTATCCTACTAGTAAGATCC, R: ATGTGTGCCTTATACTTCTCG |
| Hc18188 | (GCCA)_5_ GCC | F: TACATTCAGCCGGAATGTGAC, R: TCCGGAGTCCTACTTCATCTG |
