# Supplementary material for: Introgression of Ivermectin Resistance Genes into a Susceptible Haemonchus contortus Strain by Multiple Backcrossing
Source: PLoS Pathog. 2012 Feb 16;8(2):e1002534. doi: 10.1371/journal.ppat.1002534 (PMC3280990; doi:10.1371/journal.ppat.1002534)
Supplement: Table S2 — Treatment efficacies based on worm burden. Arithmetic mean (±SEM) and range of H. contortus counts, sex differentiation of worm burdens and percentage efficacies. (DOC) [file ppat.1002534.s005.doc]

**Table S2. Treatment efficacies based on worm burden.** Arithmetic mean (±SEM) and range of *H. contortus* counts, sex differentiation of worm burdens and percentage efficacies.

| Strain | Treatment group | Arithmetic mean total *H. contortus* count (±SEM) [range] | Worm burden percentage efficacy | Female:male ratio |
| --- | --- | --- | --- | --- |
| MHco3(ISE) | Untreated control | 2750 (±266) [2100 - 3650] |  | 0.8 |
|  | 0.1 mg/kg ivermectin | 0 (±0) [0-0] | 100 |  |
|  | 0.2 mg/kg ivermectin | 0 (±0) [0 - 0] | 100 |  |
| MHco4(WRS) | Untreated control | 3950 (±776) [1550 - 5700] |  | 1.0 |
|  | 0.1 mg/kg ivermectin | 3240 (±218) [2450 - 3650] | 18 | 0.9 |
|  | 0.2 mg/kg ivermectin | 1970 (±300) [1100 - 2800] | 50 | 0.9 |
| MHco10(CAVR) | Untreated control | 2720 (±803) [500 - 5100] |  | 0.8 |
|  | 0.1 mg/kg ivermectin | 2110 (±338) [1300 - 3000] | 22 | 0.7 |
|  | 0.2 mg/kg ivermectin | 1700 (±100) [1450 - 2050] | 38 | 1.0 |
| MHco3/10.BC_4_ | Untreated control | 1662 (±213) [970 - 2150] |  | 1.0 |
|  | 0.1 mg/kg ivermectin | 150 (±50) [20 - 300] | 91 | 1.9 |
|  | 0.2 mg/kg ivermectin | 168 (±76) [10 - 440] | 90 | 1.2 |
| MHco3/4.BC_4_ | Untreated control | 1256 (±135) [880 - 1690] |  | 1.1 |
|  | 0.1 mg/kg ivermectin | 282 (±70) [130 - 450] | 78 | 1.2 |
|  | 0.2 mg/kg ivermectin | 72 (±18) [30 - 140] | 94 | 1.4 |
