# Supplementary material for: Introgression of Ivermectin Resistance Genes into a Susceptible Haemonchus contortus Strain by Multiple Backcrossing
Source: PLoS Pathog. 2012 Feb 16;8(2):e1002534. doi: 10.1371/journal.ppat.1002534 (PMC3280990; doi:10.1371/journal.ppat.1002534)
Supplement: Table S3 — Treatment efficacies based on faecal egg count reduction. Arithmetic mean (±SEM) and range of faecal egg count and percentage efficacy seven days post-treatment. (DOC) [file ppat.1002534.s006.doc]

**Table S3. Treatment efficacies based on faecal egg count reduction.** Arithmetic mean (±SEM) and range of faecal egg count and percentage efficacy seven days post-treatment.

| Strain | Treatment group | Day 7 post treatment arithmetic mean FEC (±SEM) [range] | Day 7 post treatment efficacy | Bootstrap  treatment efficacy (95% confidence intervals) |
| --- | --- | --- | --- | --- |
| MHco3(ISE) | Untreated control | 6386 (±1713) [1386 - 11664] |  |  |
|  | 0.1 mg/kg ivermectin | 14 (±12) [1 - 63] | 100 | 100 (99, 100) |
|  | 0.2 mg/kg ivermectin | 19 (±8) [0 - 39] | 100 | 100 (99, 100) |
| MHco4(WRS) | Untreated control | 7351 (±1500) [4050 - 11358] |  |  |
|  | 0.1 mg/kg ivermectin | 9778 (±2729) [3762 - 19080] | 0 | 0 (0, 33) |
|  | 0.2 mg/kg ivermectin | 4716 (±2021) [1944 - 12510] | 36 | 34 (0, 76) |
| MHco10(CAVR) | Untreated control | 5540 (±1532) [486 - 8766] |  |  |
|  | 0.1 mg/kg ivermectin | 3245 (±273) [2520 – 3960] | 41 | 39 (0,62) |
|  | 0.2 mg/kg ivermectin | 3508 (±959) [441 - 6048] | 37 | 34 (0, 73) |
| MHco3/10.BC_4_ | Untreated control | 1302 (±436) [261 - 2637] |  |  |
|  | 0.1 mg/kg ivermectin | 143 (±22) [109 – 231] | 89 | 88 (70, 95) |
|  | 0.2 mg/kg ivermectin | 106 (±50) [18 – 279] | 92 | 91 (73, 99) |
| MHco3/4.BC_4_ | Untreated control | 4761 (±754) [3006 - 6822] |  |  |
|  | 0.1 mg/kg ivermectin | 1463 (±589) [585 - 3762] | 69 | 69 (38, 87) |
|  | 0.2 mg/kg ivermectin | 348 (± 86) [126 - 594] | 93 | 92 (88, 97) |
